# Numerical model of the locomotion of oscillating ‘robots’ with frictional anisotropy on differently-structured surfaces

**DOI:** 10.1038/s41598-024-70578-1

**Published:** 2024-08-24

**Authors:** Alexander Filippov, Stanislav Gorb

**Affiliations:** 1 Department of Functional Morphology and Biomechanics, Zoological Institute of the Kiel University, Am Botanischen Garten 1–9, 24098 Kiel, Germany; 2 Donetsk Institute for Physics and Engineering, National Academy of Science, Donetsk, Ukraine

**Keywords:** Anisotropic friction, Tribology, Surfaces, Locomotion, Robotics, Biomimetics, Animal, Plant, Computational biophysics, Bioinspired materials, Computational science

## Abstract

In engineering materials, surface anisotropy is known in certain textured patterns that appear during the manufacturing process. In biology, there are numerous examples of mechanical systems which combine anisotropic surfaces with the motion, elicited due to some actuation using muscles or stimuli-responsive materials, such as highly ordered cellulose fiber arrays of plant seeds. The systems supplemented by the muscles are rather fast actuators, because of the relatively high speed of muscle contraction, whereas the latter ones are very slow, because they generate actuation depending on the daily changes in the environmental air humidity. If the substrate has ordered surface profile, one can expect certain statistical order of potential trajectories (depending on the order of the spatial distribution of the surface asperities). If not, the expected trajectories can be statistically rather random. The same presumably holds true for the artificial miniature robots that use actuation in combination with frictional anisotropy. In order to prove this hypothesis, we developed numerical model helping us to study abovementioned cases of locomotion in 2D space on an uneven terrain. We show that at extremely long times, these systems tends to behave according to the rules of ballistic diffusion. Physically, it means that their motion tends to be associated with the “channels” of the patterned substrate. Such a motion is more or less the same as it should be in the uniform space. Such asymptotic behavior is specific for the motion in model regular potential and would be impossible on more realistic (and complex) fractal reliefs. However, one can expect that in any kind of the potential with certain symmetry (hexagonal or rhombic, for example), where it is still possible to find the ways, the motion along fixed direction during long (or even almost infinite) time intervals is possible.

## Introduction

Mechanical systems using anisotropic surfaces are prevalent in the technical world, spanning from the molecular level to the macroscopic scale. In engineering, anisotropy is deliberately created in certain textured patterns of polycrystalline materials, either naturally or artificially during the manufacturing process. Geological structures also exhibit anisotropy on a large scale due to tectonic processes. In biological systems, many surfaces are covered with micro- and nanostructures oriented at specific angles to the supporting surface, resulting in mechanical anisotropy^1–13^. These structures lead to different frictional and mechanical interlocking properties during sliding contact in various directions^14–18^. Such surfaces play a role in generating propulsion on the substrate or within the substrate for purposes such as locomotion^19–24^ or transporting items^25–28^. Mechanical systems with anisotropic surfaces are found in diverse organisms, including insect unguitractor plates^29–31^, interlocking mechanisms in insect legs and antennae^7^, insect ovipositor valvulae^5,32^, animal attachment pads^7,16,18^, inner surfaces of pitcher plants^11,12,33^, wheat awns^17^, fluid-transporting systems in plants^28^, butterfly wings^34^, and more.

These diverse examples serve various functions, ranging from the transportation of particles (as observed in cleaning devices)^15,25–27^ to the positioning of leaves atop one another^13^ and even in generating propulsion during slithering locomotion, as seen in snakes^35,36^. Considering the potential influence of the rigidity of the support on the mechanical behavior of these systems, as demonstrated in recent studies on snake skin^37^, and using previous theoretical approaches from the field of tribology^38,39^ we have previously developed a model explaining the anisotropic friction efficiency by examining the following factors: (1) the slope of the surface structures, (2) the rigidity of their joints, and (3) sliding speed^24^. Through the proposed model, we put forth a generalized optimal set of variables aimed at maximizing the functional efficiency of anisotropic systems of this nature. We also explored the optimal combination of these parameters from the perspective of biological systems.

**Fig. 1:**
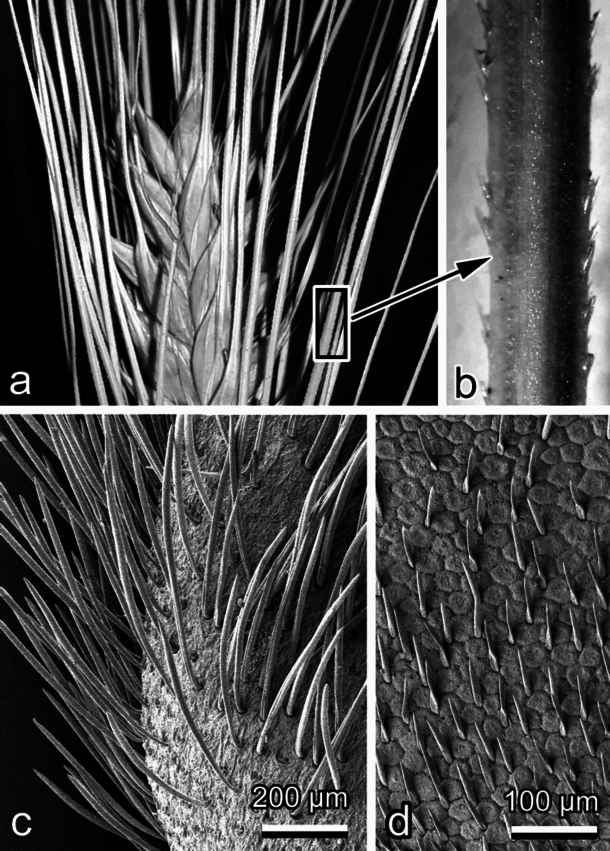
Biological systems, where frictional anisotropy supports directional motion. (**a**,**b**). Wheat awns (with minute spines) capable of self actuation using anisotropically oriented cellulose fibres. (**c**). Diaspores of the plant *Bituminaria bituminata* (Fabaceae) covered by minute trichomes and capable of directed sliding under the action of external forces (wind, vibrations, etc.). (**d**). Surface of the maggot of the black soldier fly *Hermetia illucens* (Diptera, Stratiomyidae) using microscopical setae to generate directed propulsion in contact with substrates.

However, as mentioned above, in biology, there are numerous examples of mechanical systems which combine anisotropic surfaces with the motion, elicited due to some actuation using muscles (as it is the case in worms or worm-like larvae of insects, Fig. 1a,b) or by stimuli-responsive materials, such as highly ordered cellulose fiber arrays of plant seeds (Fig. 1c,d). The first ones are rather fast actuators, because of the relatively high speed of muscle contraction. The latter ones are very slow, because they generate actuation depending on the daily changes in the environmental air humidity. In any case, such systems can generate motion in 2D space and the trajectories of such systems will depend on the surface microstructure of the substrate.

If the substrate has ordered surface profile, one can expect certain statistical order of potential trajectories (depending on the order of the spatial distribution of the surface asperities). If not, the expected trajectories can be statistically rather random. The same presumably holds true for the artificial miniature robots that use actuation in combination with frictional anisotropy^40–43^. In order to prove this hypothesis, we developed numerical model helping us to study abovementioned cases of locomotion in 2D space on the uneven terrain. In previous publications, we studied numerous natural systems that demonstrate random motion on random terrains, generating self-organized patterns the motion. In each particular case these patterns and scenarios of their formation depend on the system under consideration, interactions and rules of game involved into the model. The present study is devoted to qualitatively different, specific case of the system. Here source of the locomotion is basically produced inside of each individual and supported by the frictional anisotropy. These both features in combination with the substrate structure produce interesting patterns of trajectories’ distribution, which are not necessarily easily predictable from the very beginning. It is caused by an appropriately chosen combination of oscillations of internal degree of freedom and its friction anisotropy from head to tail along the body of an object (robot, automaton).

## Numerical model

Basically, the model consists of two components: (1) The oscillating ‘robots’ (according to our previous works, we will call moving objects of the model “*movable digital automata*”) and (2) potential 3D surface relief, on which these automata are moving.

Below, to get fast accumulation of the statistical data we will use a large number of the same automata simultaneously, but in contrast to the majority of our previous works^44,45^, they will not interact with each other, but move independently from the neighbours. Such an approach, using high number of simultaneous realizations, was motivated in this study by an immediate observation that even for very simple substrate potential, the motion of sole automaton demonstrates, in general case, rather complex and practically unpredictable behavior. It depends on many factors, including randomly chosen initial position, frequency and amplitude of its oscillations and length of the automaton “body” connecting opposite ends (“head” and “tail”) of the robot.

The behavior of the robot depends also on the amplitude and characteristic spatial scale (period, if it exists) of the substrate potential, mutual relation and the friction anisotropy of both the “head” and “tail”. Let us mention that in this particular study they are necessarily different. Generally speaking, there are different masses on the both sides as well. Counting on this, dynamic scenario essentially depends on an initial orientation of the “body” relatively to the substrate.

This, minimal in fact, list of the parameters certainly means that it is practically impossible to simulate all the allowed combinations of the parameters one after another. Even if such sequence of the numerical experiments is hypothetically done, it would be impossible to visualize all of them in dynamics and collect a set of static pictures and curves with the results. At the same time, it is important to agree on some general procedure, which allows generation of the results more or less systematically, and to select some most important “classes” of possible scenarios. Despite of the quantitative deviations of the behavior, it limits the number of relatively general qualitatively different realizations of the complete system and its behavior. Below we illustrate this by the cases that seem to be important to our opinion.

**Fig. 2:**
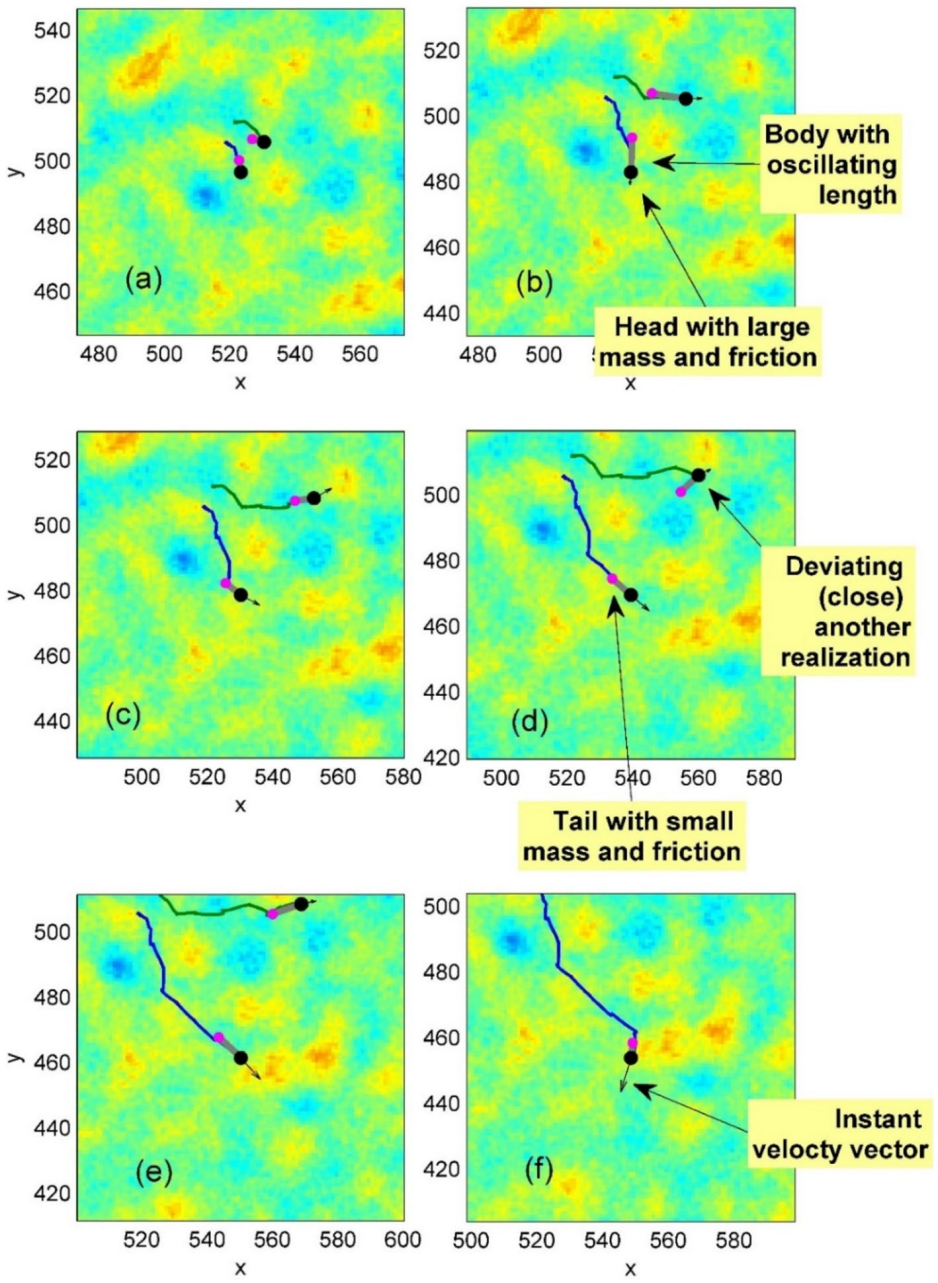
Numerically generated potential and basic properties of individual movable automata (see also main text). Heavy “head” with high friction is shown by the large black circle. Small pink circle represents light “tail” with much smaller friction. These two ends are connected by a gray line (“body”) with oscillating length. Due to the difference in mass and friction coefficient $m_{1} > m_{2}$,$\gamma_{1} > \gamma_{2}$, at every oscillation cycle, the common center of mass shifts into the direction from the tail to head. Potential relief (shown by the colormap) causes additional forces, which lead to the change of the motion direction. As a result, a complex curved trajectory appears. In the complex potential relief, trajectories of two originally close realizations of the system mutually deviate. See supplementary Movie 1.

One can prove that from the computational time point of view, there is not strong difference between 2- or 3-dimentional spaces, where the motion of the automaton takes place. However, it is much easier to visualize the results in 2d case. Thus, for definiteness and simplicity, in this paper, we limited ourselves by two-dimensional case only. In this case, one can treat the space as natural potential relief. Typically, such a relief contains two components: universal one, with so-called fractal distribution of the maximums and minimums having scale-invariant amplitudes and wavelengths and the having one or more kinds of the peculiarities having specific scales of the potential extremums. Such a potential can be numerically generated by the procedure described in a number of our previous publications^46–52^ and depicted by the surface or colormap plots analogous to the one shown in Fig. 2 as an example (here the standard Matlab colormap ‘jet’ is used).

Briefly, the potential generation can be described as follows. The majority of real surfaces of the substrates have semi-fractal structure with some Fourier spectrum and amplitude of roughness. It is well accepted in the literature that such surfaces are self-affine fractal given by the real part of the following function:

1 $$ Z(x,y) = A\iint {dq_{x} dq_{y} C(q)\exp (iq_{x} x + iq_{y} y + \zeta (x,y))}, $$

with scaling spectral density $C(q)$. Here $A$ is the amplitude of the surface roughness,$i$ is imaginary unit, $q_{x,y}$ are the Fourier components along $x$ and $y$ directions with an absolute value $q = \sqrt {q_{x}^{2} + q_{y}^{2} }$, and $\zeta (x,y)$ is $\delta$-correlated random phase $< \zeta (x,y) > = 0$;$< \zeta (x,y)\zeta (x^{\prime},y^{\prime}) > = D\delta (x - x^{\prime})\delta (y - y^{\prime})$. The wave-vectors vary from the minimum $q_{\min }$ to the maximum $q_{\max }$ defining an interval of the scales, where semi-fractal structure of the surface may take place.

Details of the generation procedure for the profile $Z(x,y)$ have been described in abovementioned previous papers. In many cases, the surface is visually (macroscopically) flat, but in the current literature^53^, it is accepted that for the majority of such physical surfaces the expansion Eq. (1) has scale-invariant spectral density $C(q) = 1/q^{\beta }$ with the exponent $\beta \approx 0.9$. However, here, as in many other biologically-related problems, the surface is not so simple and general. The surface $Z(x,y)$ has actually some characteristic scale(s) of the peculiarities with at least one selected wave-vector $q_{0}$. To include such property of the surface into consideration, one has to combine its spectral density from the two terms $C(q) = \mu C_{1} (q) + \nu C_{2} (q)$ reproducing both different impacts into the surface. The first term, as before, the scaling part of the surface $C_{1} (q) = 1/q^{\beta }$ and the second one $C_{2} (q) = \exp \left\{ { - [(q - q_{0} )/\Delta q]^{2} } \right\}$, which describes a part having minimums randomly distributed around the selected wave-vector $q_{0}$ with some width $\Delta q$. The coefficients $\mu$ and $\nu$ determine different weights of the corresponding substructures in the total surface $Z(x,y;q_{0} )$. Below, according to our preliminary works and for the definiteness, we use: $\Delta q = 3q_{\min }$, $\mu = 0.2$ and $\nu = 1$.

At every fixed value of $q_{0}$, each formal calculation of the surface $Z(x,y;q_{0} )$ according to Eq. 1 generates a new variation of the function $Z(x,y;q_{0} )$. It is distributed between the particular global minimum $Z_{\min }$ and global maximum $Z_{\max }$. To describe all the realizations of the surface $Z(x,y;q_{0} )$ in regular manner, it is convenient to normalize it onto the difference $Z_{\max } - Z_{\min }$ and multiply it by a scalar factor $U_{0}$: $U(x,y;q_{0} ;U_{0} ) = U_{0} Z(x,y;q_{0} )/(Z_{\max } - Z_{\min } )$, which will define actual amplitude of roughness. Final combination $U(x,y;q_{0} ;U_{0} )$ plays a role of an effective potential in the equations of motion depending on both physically important parameters: amplitude $U_{0}$ and statistical properties of the surface characterized by the wave-vector $q_{0}$.

Let us now write the equations of motion of each particle which make up an automaton in general form of the Newtonian equations including potential forces caused by the relief as well as by the energy dissipation:

2 $$ \begin{gathered} m_{k} d^{2} x_{k} /dt^{2} - \gamma_{k} dx_{k} /dt = - \partial U(x_{k} ,y_{k} ;q_{0} ;U_{0} )/\partial x_{k} + f_{k,x}^{ext} \hfill \\ m_{k} d^{2} y_{k} /dt^{2} - \gamma_{k} dy_{k} /dt = - \partial U(x_{k} ,y_{k} ;q_{0} ;U_{0} )/\partial y_{k} + f_{k,y}^{ext} ,\,k = [1,\,\,2]. \hfill \\ \end{gathered} $$

Here $f_{x}^{ext}$ and $f_{y}^{ext}$ are *x*- and *y*-components of the “external force”, which is generated by the oscillations of the body of an automaton. Anisotropic friction is caused by the difference in friction coefficients, dissipation and masses of its “head” and “tail”, which are described below by the indices 1 and 2, respectively. Below, we suppose that $m_{1} > m_{2}$ and $\gamma_{1} > \gamma_{2}$. Different local energy dissipation on different sides of the movable automaton causes its motion forward.

Oscillations and anisotropic friction of the automata are organized as follows. We connect to ends of the automaton an elastic “body” with an equilibrium length: $l{}_{0} = const$. Deformation of this length causes an elastic force with the components

3 $$ \begin{gathered} f_{y}^{elastic} = f_{0}^{elastic} (x_{2} - x_{1} )[1 - (l(x_{1} ,x_{2} ,y_{1} ,y_{2} ;t)/l_{0} )^{2} ] \hfill \\ f_{y}^{elastic} = f_{0}^{elastic} (y_{2} - y_{1} )[1 - (l(x_{1} ,x_{2} ,y_{1} ,y_{2} ;t)/l_{0} )^{2} ], \hfill \\ \end{gathered} $$

and vary the distance between the ends around the equilibrium one with a frequency $\omega$:$l(t) = l_{0} [1 + dl\cos (\omega t)]$. The forces (Eq. 3) tend to return elastically length of the body $l(x_{1} ,x_{2} ,y_{1} ,y_{2} ;t) = \sqrt {[x_{1} (t) - x_{2} (t)]^{2} + [y_{1} (t) - y_{2} (t)]^{2} }$ to its current “equilibrium” value, which is varied by itself with time $l(t)$, which, however, changes with the time.

If the masses and dissipation constants of both sides of the automaton are equal, it should oscillate around the center of mass: $x_{c} = (m_{1} x_{1} + m_{2} x_{2} )/(m_{1} + m_{2} )$. However, if they are different, the behavior is not so trivial. For example, it is quite predictable, that if one of the constants is much larger than another, the body on this end (below we call it “head”) will be damped during the extension stage of the oscillation period. It will practically stop, in its present position. However, the length of the body forcibly tends to return to its value $l(t) = l_{0} [1 + dl\cos (\omega t)]$. Because of this, another, more mobile part of the system, should move into direction of the damped one. In the next period of the oscillation, the length is forcibly extended again, and both sides move apart from new position of the center of mass.

The main features of the movable automaton and potential mentioned above are written directly above the subplots of the conceptual Fig. 2. Each subplot illustrates instant positions and trajectories of minimal number (two) initially originally close realizations, plotted as stroboscopic stages with fixed time step $\Delta t$ between them. The intervals for the visualization $\Delta t$ can be chosen arbitrary, but in any case, they should be larger than microscopic time step numerical simulation $dt < < \Delta t$, where in turn $dt < < 1$. Heavy “head” with high friction is shown by the large black circle. The small pink circle represents light “tail” with much smaller friction. These two ends are connected by a gray line (“body”) with oscillating length. Due to the difference in the masses and friction coefficients $m_{1} > m_{2}$,$\gamma_{1} > \gamma_{2}$, at every oscillation cycle, the common center of the masses shifts into the direction from the tail to head.

Potential relief (shown by the colormap) causes additional forces, which lead to the changes of the motion direction. As a result, a complex curved trajectory appears. In the complex potential relief trajectories of two originally close realizations of the system deviate (see Fig. 2). As a result, during each cycle of extension-compression, the common center of mass (in fact, the whole system) shifts into direction from the tail to head. At the first glance, it looks paradoxically. However, the system does not violate the laws of mechanics, because an anisotropic damping actually corresponds to the different interaction with the stationary fixed substrate in a direction from the tail to head of the system. In this paper, we do not discuss different physical realizations of such a system. However, the model proposed here gives us a solid foundation allowing for testing a plenty of different parameter combinations.

It is especially meaningful, because even being generally understood, the effect of the automaton propagation is essentially complicated in the presence of substrate and depending on its particular structure. Both ends of the automaton tend to return into the center along straight line in an isotropic space only. In reality, each of them feels both force components $- \partial U(x_{k} ,y_{k} ;q_{0} ;U_{0} )/\partial {\mathbf{r}}_{k}$ defined independently in two different places of the potential surface $\partial U(x_{k} ,y_{k} ;q_{0} ;U_{0} )$. In other words, besides to the propulsion, the body connecting the head and tail rotates. It is one of the main reasons, why we use an elastic connection between them (Eq. 3). Otherwise, the standard description, based on solving mechanical equations of motion for rotating rigid many-body system with varied lengths of its segments, would cause rather complex time consuming problem for an extremely large (after all) number of realizations.

Initially, each robot (automaton) starts to move in a randomly chosen direction. Direct numerical solution of the equations of motion Eq. (2) shows that its trajectory and the entire “history of events”, which happens to the automaton, strongly depends on the initial direction, frequency of oscillations, frictions coefficients, etc. Considering the initial conditions for the system Eq. (2), one can remind, that each time the potential specified in Eq. (1) is randomly generated. Thus, even at fixed parameters $U_{0}$ and $q_{0}$, one deals with a new realization of the surface $Z(x,y)$ and with corresponding effective potential $U(x,y;q_{0} ;U_{0} )$. Because of this, one has really a cloud of the realizations with trajectories strongly deviating one from another.

Typical family of the trajectories of movable automata starts from an area marked by white rectangle on the randomly generated potential relief. Instant positions of the “heads” and “tails” of each automaton are marked by the large and small black circles respectively. Instant velocities are represented by the vector arrows. One has to notice that the directions of the velocities do not coincide with the direction of each automaton body from its “tail” to the “head”. Generation of an analogous configuration in dynamics is reproduced in the supplementary Movie 1.

**Fig. 3:**
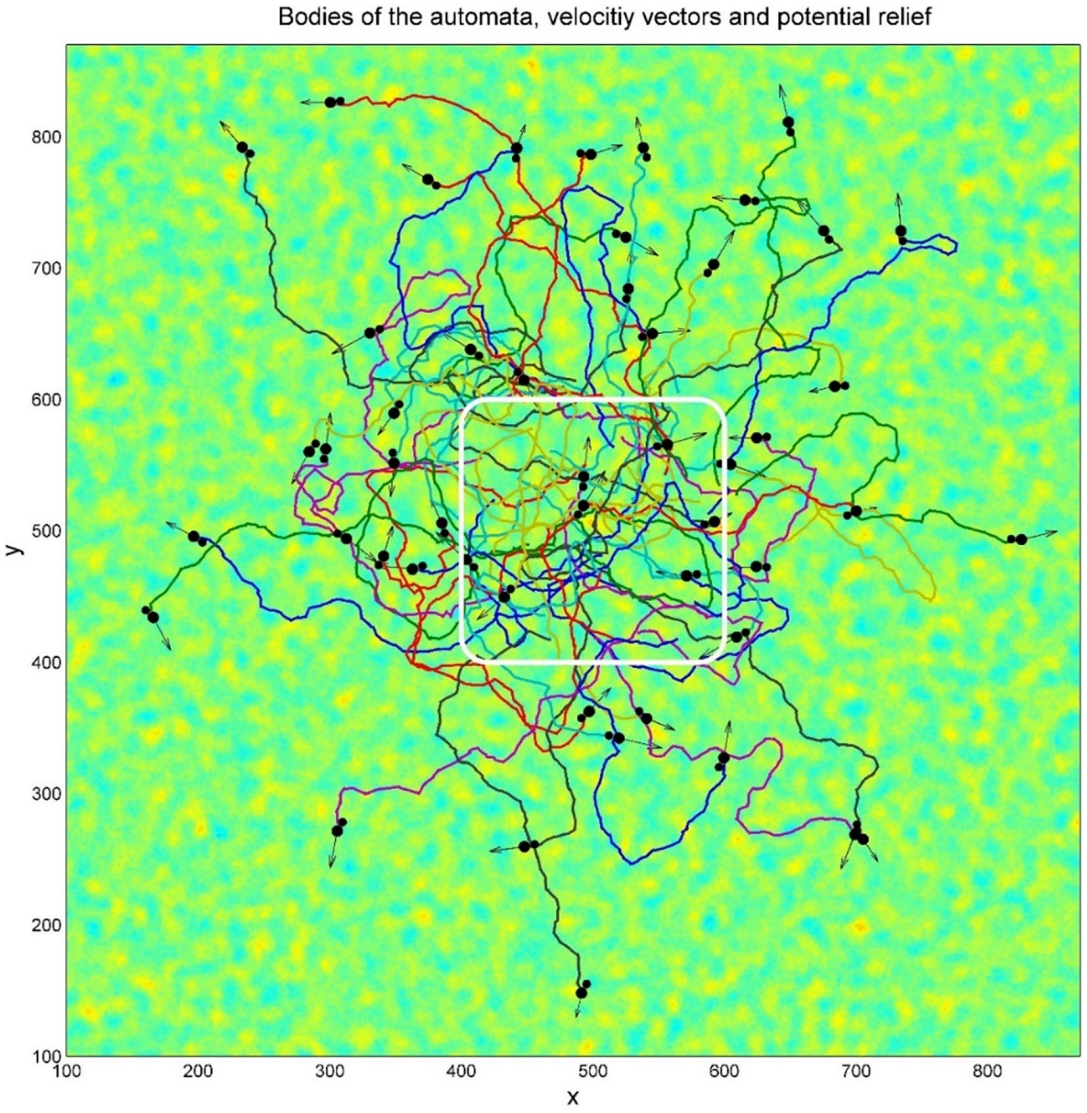
Typical family of the trajectories of movable automata starting from an area marked by the white rectangle on the random potential relief. Instant positions of the “heads” and “tails” of each automaton are marked by the large and small black circles respectively. Instant velocities are represented by the vector arrows. The directions of the velocities do not coincide with the body direction of each automaton.

**Fig. 4:**
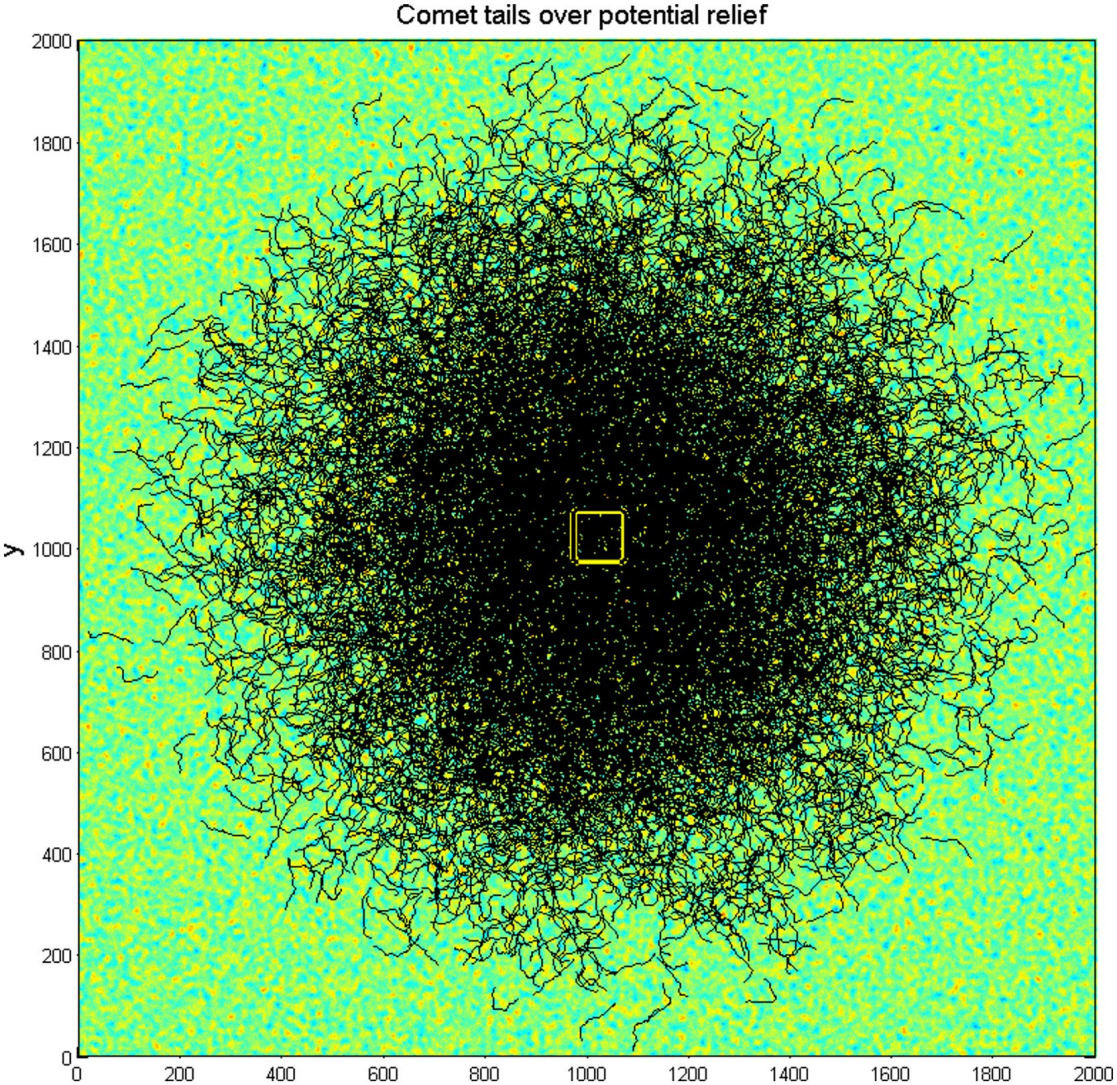
Large scale structure of the “comet tails” reproducing the automata trajectories found during an interval $0 < \tau < t$ from some preliminary moment $t - \tau$ to the present one $t$. Starting from a point ${\mathbf{r}}_{j} (t - \tau ) = \{ x_{j} (t - \tau ),y_{j} (t - \tau )\}$ every automaton with a number $j = 1,2,...N$ can move in an arbitrary direction on the complex potential relief. One can see numerous returns of the trajectories to densely populated central area. Central region, where the initial conditions of all the trajectories are located, is marked by the yellow rectangle.

Complex structure of the expanding cloud of the realizations is seen already for a relatively small number of automata as reproduced in Fig. 3. However, the statistical structure of the cloud can be found only for sufficiently large number of realizations. Figure 4 illustrates this for $N = 10{}^{4}$ of them. In particular, it shows a typical tool, which we will use below. If the number of the trajectories is too large, the picture becomes nontransparent and it is not possible either to separate visually the trajectories one from another or observe a correlation between the density of curves and the structure of potential. Therefore, in order to do this, we plot relatively short fragments of the trajectories (so called comet-tails), which allow for conserving visual transparency of the picture to some extent.

**Fig. 5:**
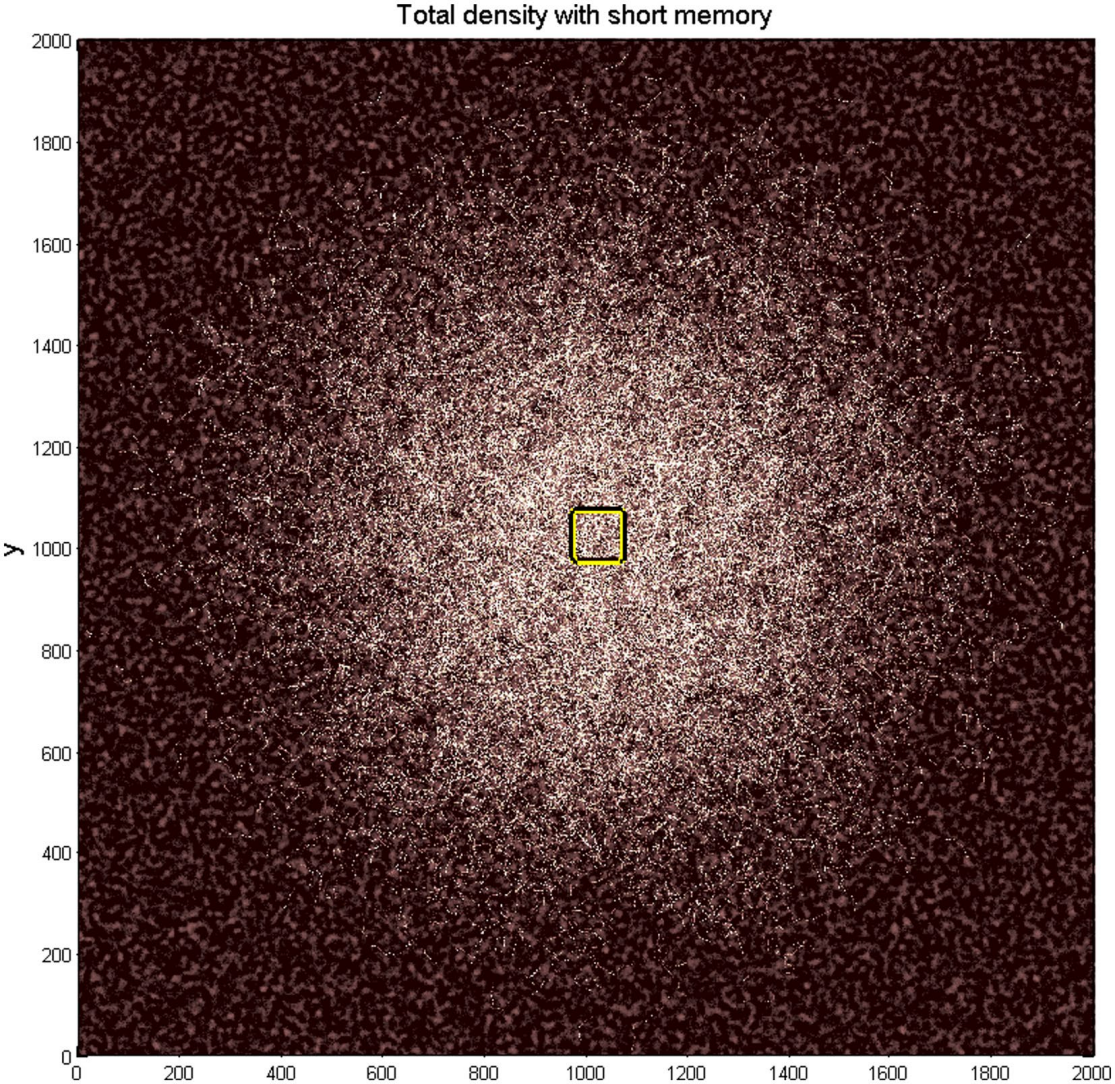
Density of the trajectories accumulated with a short-time memory for the same number of the independent automata realizations $N = 10{}^{4}$ during the same procedure as depicted in Fig. 4. Higher local density corresponds to the brighter regions of the colormap. Complexity of the density distribution reflects abovementioned combination of two complexities: (1) the surface relief and (2) the dynamic motion of the oscillating automata with anisotropic friction. Simultaneous generation of the Figs. 3 and 4 is reproduced in dynamics in the supplementary Movie 2.

The same observation about the demanded density of the realization cloud is correct in relation to the formally accumulated total time, which all the realizations spend in every place of the substrate (“density of states”). To get more or less transparent picture about the sequence of events, one has to do it with the continuous loss of information about previous states of the system. We present such a result in Fig. 5, where the density of trajectories was accumulated with a short-time memory for the same number of the independent automata realizations $N = 10{}^{4}$ during the same procedure, as depicted in Fig. 4. Higher local density corresponds to the brighter regions of the colormap.

**Fig. 6:**
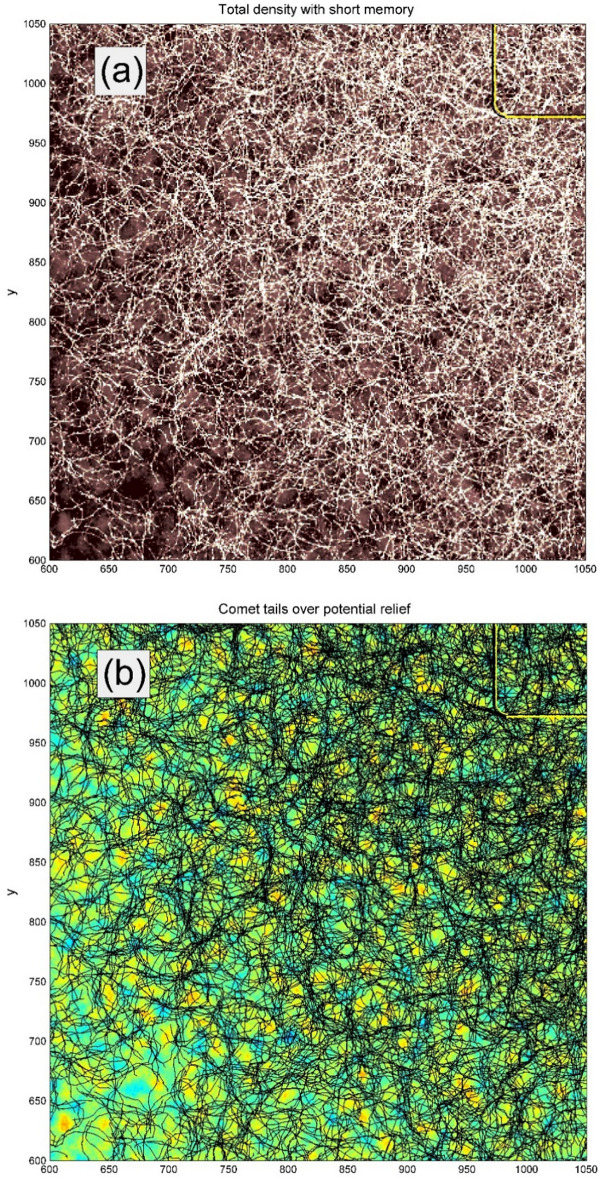
Enlarged fragments of Figs. 3 and 4 (subplots (**a**) and (**b**)) demonstrating mutual fine correlations between accumulated densities and the trajectories plotted over substrate potentials. Part of the yellow rectangle marks the region, where the motion of automata started (the region is, however, almost outside of this plot).

Complexity of the density distribution reflects abovementioned combination of the complexities of the surface relief and dynamic motion of the oscillating automata with anisotropic friction. More transparent enlarged fragments of Figs. 4and 5 are given in the Fig. 6 (subplots (a) and (b)) demonstrating mutual fine correlations between accumulated densities and the trajectories plotted over substrate potentials. Simultaneous generation of the Figs. 4 and 5 is reproduced in dynamics in the supplementary Movie 2.

Considering the found complexity of this problem on the realistic substrates, we decided to reduce its initial complexity to the idealized case of regular periodic relief. Let us start with the potential having square symmetry. On one side, it conserves main properties of the system caused by anisotropy of the space (substrate). On the other side, the square symmetry allows for more or less clear separation of the effects appearing due immanent properties of the oscillating anisotropic movable automata and shadowing them symmetry of the static system.

**Fig. 7:**
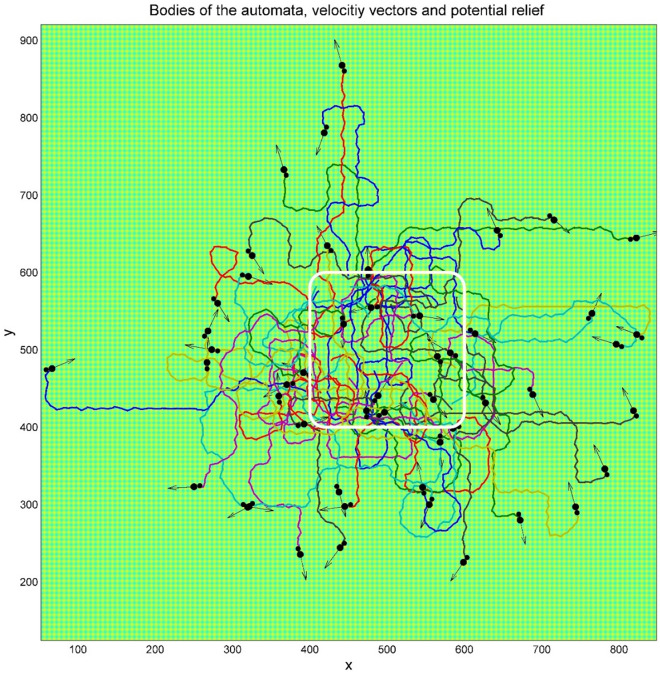
The trajectories same as in Fig. 2, obtained in the case of potential relief having simple square symmetry. One can notice the trajectories corresponding to the particular realizations which move during long time periods along almost fixed direction, determined by symmetrically defined (but arbitrary selected) perpendicular directions on the substrate having square symmetry.

Such an advantage of the simple relief is immediately seen from Fig. 7, where the same system with relatively small number of realizations, as in Fig. 2, but obtained in potential relief with simple square symmetry, is shown. The main advantage is that one can directly notice the trajectories corresponding to the particular realizations which move for a long time along fixed directions determined by the selected (perpendicular) directions of the potential with the square symmetry.

**Fig. 8:**
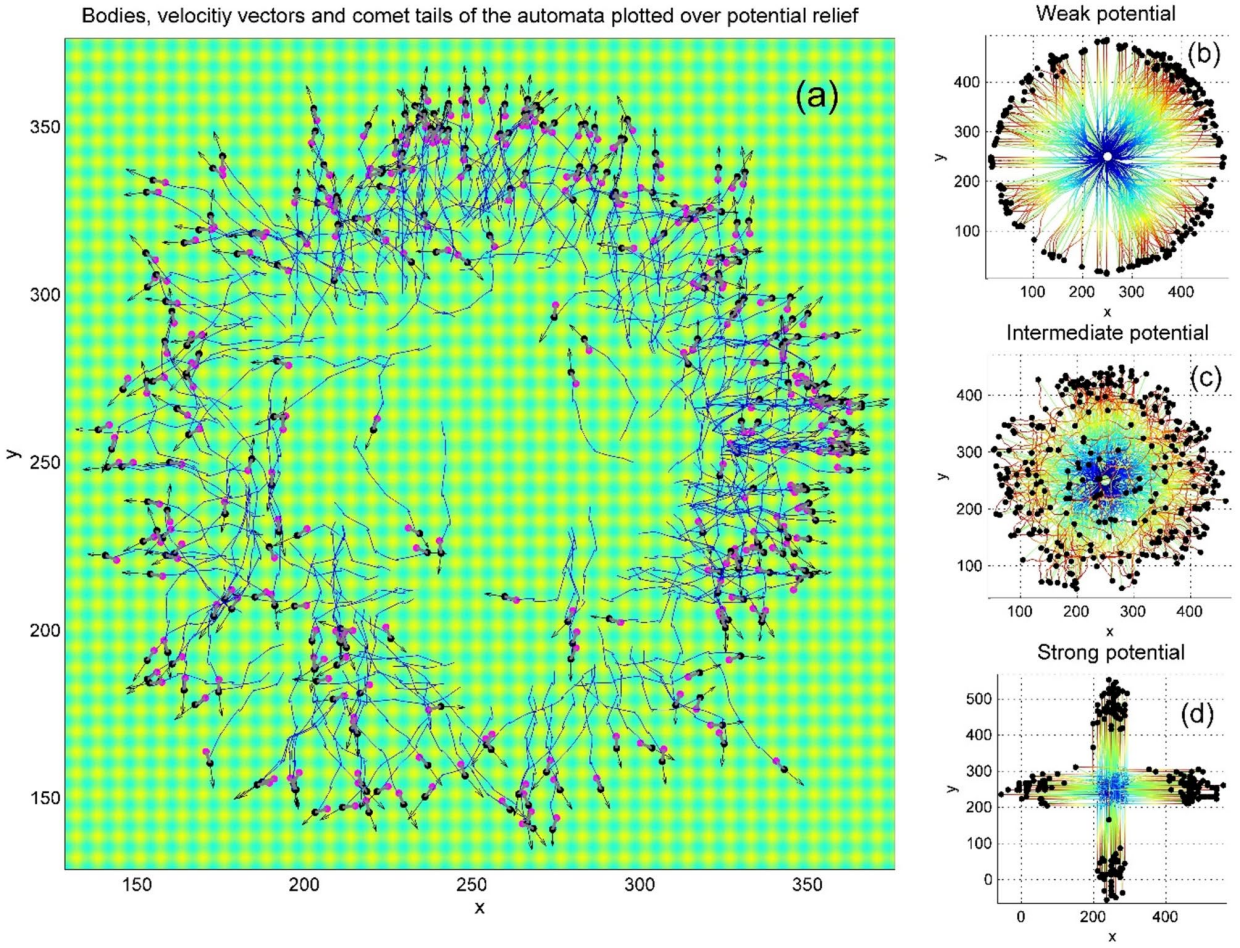
The subplot (**a**) shows an instant distribution of the automata, their velocities and comet tails trajectories found at time moment $t_{1} > \tau$ starting from a common center with random (uniform in average) distribution of the initial body orientations. The subplots (**b**), (**c**) and (**d**) show the positions of the heads and the trajectories, obtained at relatively late time moment $t_{2} > > t_{1}$ for the weak, intermediate and strong potentials, respectively. The trajectories in these subplots are colorized by the time elapsed from the beginning of motion (later time moments correspond to the red colour and earlier to the blue one, respectively). Red fragments of the trajectories, which cover the blue ones in the central part of the subplot (**c**), correspond to the returns of the automata into the area of their origination, which happened at late stages of the motion (see also Supplementary Movie 3).

We applied this advantage to investigate maybe the mostly important from practical point of view dependence of the general behavior of the system on surface potential amplitude. For different amplitudes we allowed the process to develop up to relatively late time moment and compared the patterns and results obtained. Serial generation of the configurations for different potential amplitudes is reproduced in dynamics in the Movie 3. Figure 8 reproduces three qualitatively different variants in a static form. The subplot (a) in this figure shows as an example the typical instant distribution of the automata, vectors of their velocities and comet tails of individual trajectories found at intermediate amplitude of the potential. Starting at time moment $t_{1} > \tau$ isotopically in average from a common center with random (uniform in average) distribution of the initial body orientations for an intermediate deepness of the potential transforms into, generally speaking, anisotropic one.

The subplots (b), (c) and (d) of Fig. 8 show the positions of the heads and automata trajectories, obtained at relatively late time moment $t_{2} > > t_{1}$ for the weak, intermediate and strong potentials, respectively. The trajectories in these subplots are colorized by the time elapsed from the beginning of motion. The later time moments correspond to the red and earlier to the blue parts of the spectrum, respectively. Red fragments of the trajectories, which cover the blue ones in the central part of the subplot (c) reproduce the returns of the automata to their original positions at later stages of motion. Some of such trajectories are seen also in the subplot (a).

**Fig. 9:**
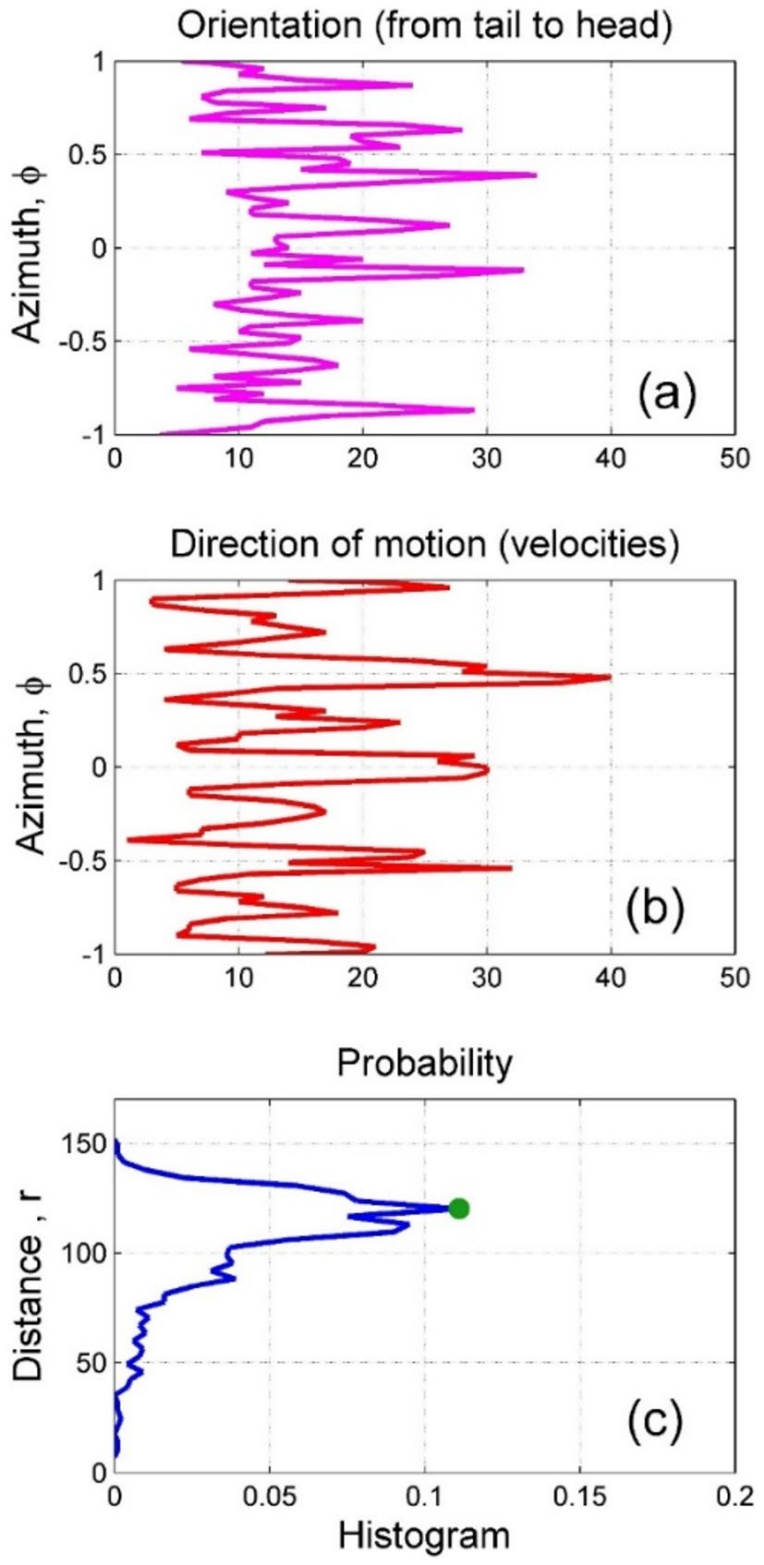
Distributions of the body orientation of automatain space (**a**) and directions of their motion obtained after long time run in the intermediate potential (**b**). Distribution of the distances between current head positions and their initial positions is plotted as a probability histogram (**c**). See also Supplementary Movie 4.

One can see directly from the movie and static screenshots that with time any automaton can find a trajectory along the perpendicular channels (normally closest to the particular realization). After this, it often continues its motion along the corresponding valley rather long time, or even forever. This observation can be supported by the statistical study of the process under consideration. To do this, let us accumulate histogram of the angles characterizing velocity direction. Individual orientation is another value, which also characterizes anisotropy of the motion. Generally speaking, these directions do not coincide. It is expected that they are somehow correlated, but instantly, the body of the selected automaton can be oriented in one direction, but its instant velocity in another one (Fig. 9).

**Fig. 10:**
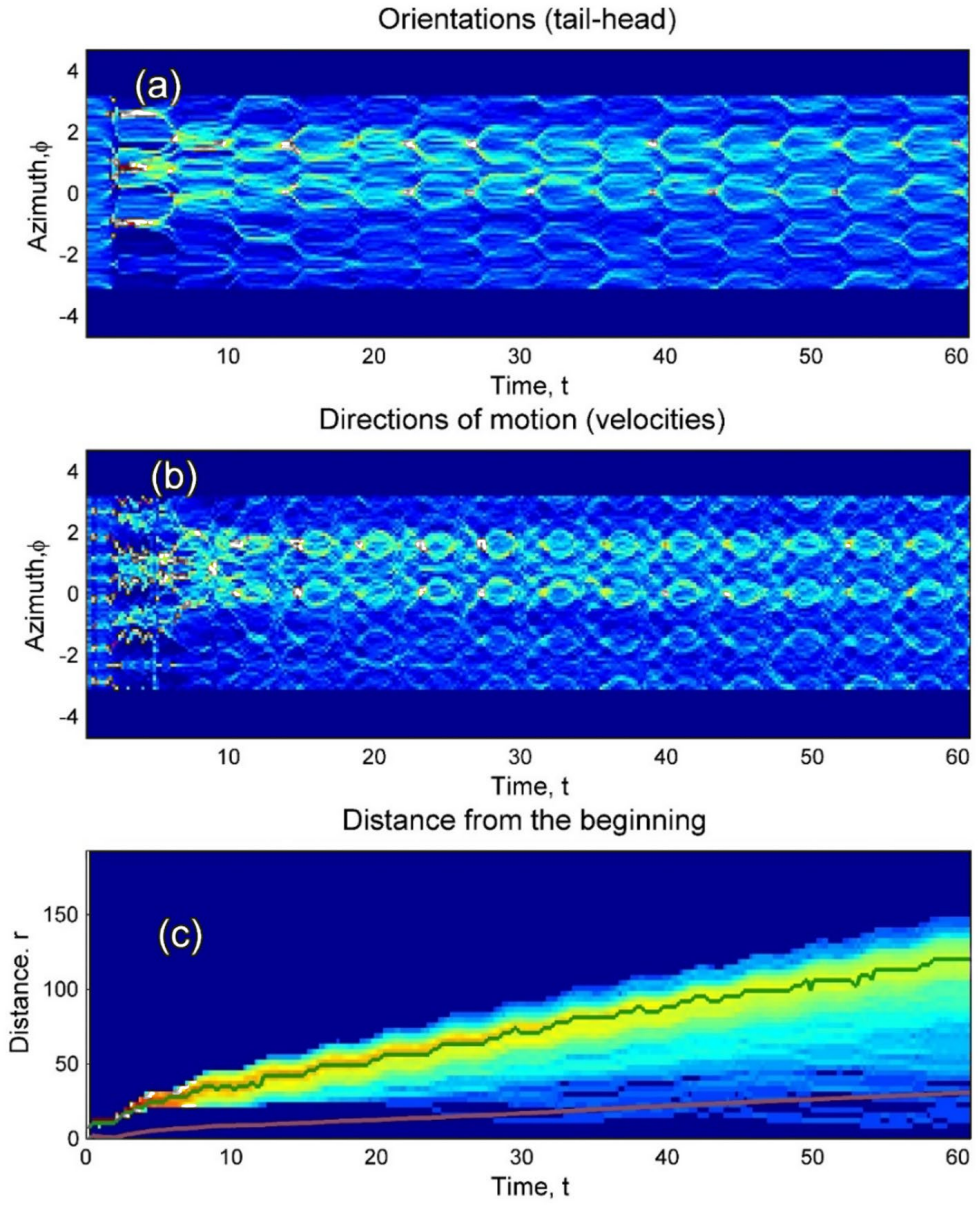
Colormaps of the time evolution of the probability histograms automata (bodies orientation in space and directions of their motion, as well as distance from the beginning in the subplots (**a**–**c**), respectively) accumulated during sufficiently long time run in the intermediate potential. The histograms, plotted in the previous Fig. 9, correspond to the last time moment. The statistical properties of the distance distribution are represented by the time-dependence of the position of probability maximum (see Fig. 9c) and characteristic width (standard deviation) of the distribution, plotted here, in the subplot (**c**), by the bold green and red curves, respectively. See also Supplementary Movie 4.

The process visually reminds diffusion, but as it is seen from its description above, it is not really diffusion, at least not in its common sense. However, expansion of the cloud of realizations in 2D space presented here still can be described in the terms, usually applied to the “diffusion” phenomena. An effective radius of the cloud can be associated with mostly dense part of the more or less isotopic front in space. Let us formally calculate a distance of each realization from the original point at given time moment and plot the histogram of distance distributions. Standard approach to diffusion problem is based on the calculation of mean square displacement (MSD). The subplot (c) in Fig. 10 can be associated with MSD. In particular, the problem can be reduced to “ordinary” MSD for random walk for a solitary realization, starting from some initial position. Here we deal with a “cloud” of the realizations which radius expands in space and simultaneously the width of expanding belt of the realizations grows. It is convenient to apply directly already existing results presented in Fig. 10 to characterize effective diffusion in the framework of present study. To avoid a misunderstanding we utilize both values: time-dependence of the position of probability maximum and characteristic width (standard deviation) of the distribution, plotted here, in the subplot (c), by the bold green and red curves.

**Fig. 11:**
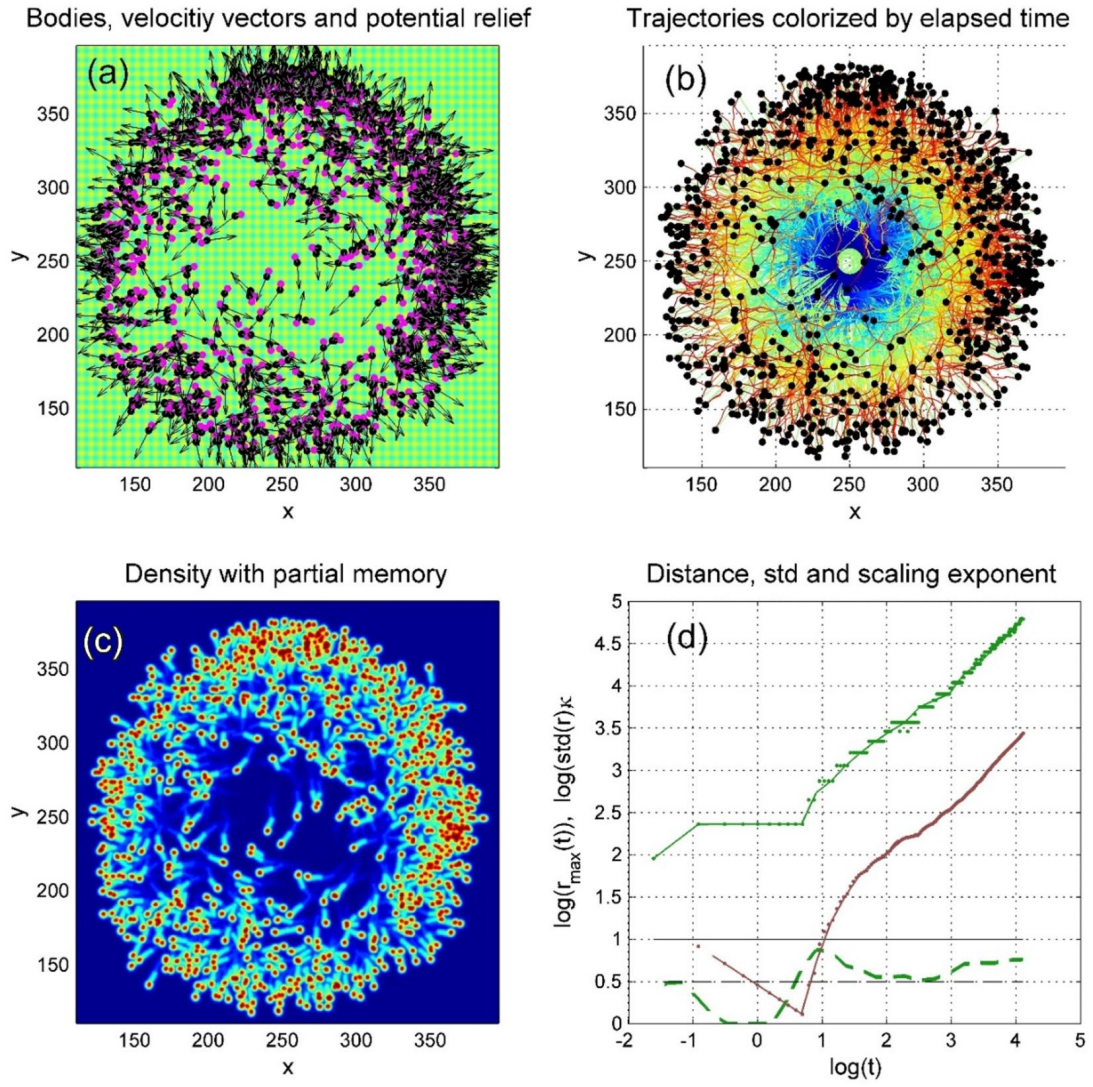
Mutual correlation between evolution of the spatial distribution of the movable automata with random initial distribution of the orientations and statistical properties of the diffusion at different stages of the process. Instant distributions of the automata, their trajectories and accumulated density are shown in the subplots (**a**–**c**), respectively. See also Supplementary Movie 4.

The density, shown in the subplot (c) of Fig. 10, is integrated over the azimuth angles. Instant realization of such a histogram is presented in the subplot (c) of Fig. 9. Due to the difference in average radial component of the velocity and especially because of the returns of some of the automata back to the proximity around the initial point, the width of this histogram increases with time. Nevertheless, rather pronounced maximum of the distribution conserves up to the sufficiently long time. Generally, it corresponds to the expanding “red belt” in Fig. 11c. Different subplots in this figure show respectively instant distributions of the automata (a), their trajectories (b) and accumulated density (c), respectively (Fig. 11a-c).

Taking into account that both these directions change in the course of motion and any instant histogram does not contain the complete information, it is useful to calculate the histograms starting from the beginning of the process and record the data. It is done in Fig. 10, where this information is presented in form of the time-angle maps for the distributions under consideration and also depicted in the subplots (b) and (a), respectively. One can see from the colormaps that after some transient period, both distributions are attracted to some stable (stationary) process, which periodically oscillates further. The curves, shown in Fig. 9a,b, correspond here to the final line of the colormap on the right end of the maps recorded at last moment $t = t_{\max }$ shown in the map. It’s why we plot the histograms in Fig. 9 in horizontal orientation.

The process visually reminds diffusion, but as it is seen from its description above, it is not really diffusion, at least not in its common sense. However, expansion of the cloud of realizations in 2D space presented here still can be described in the terms, usually applied to the “diffusion” phenomena. An effective radius of the cloud can be associated with mostly dense part of the more or less isotopic front in space. Let us formally calculate a distance of each realization from the original point at given time moment and plot the histogram of distance distributions.

Actually, it is a density, shown in the subplot (c) of Fig. 10 integrated over the azimuth angles. Instant realization of such a histogram is presented in the subplot (c) of Fig. 9. Due to the difference in average radial component of the velocity and especially because of the returns of some of the automata back to the proximity around the initial point, the width of this histogram increases with a time. Nevertheless, quite pronounced maximum of the distribution conserves up to the sufficiently long time. Generally, it corresponds to the expanding “red belt” in Fig. 11c. Different subplots in this figure show respectively instant distributions of the automata (a), their trajectories (b) and accumulated density(c), respectively (Fig. 11a–c).

Effective (time depending) diffusion exponent is defined by the relation $r_{\max } = r_{0} t^{{\kappa_{eff} (t)}}$, or in logarithmic scale by the time-depending derivative $\kappa_{eff} (t) = \partial \log (r_{\max } )/\partial \log (t)$, depicted by bold dashed green curve in the subplot (d). The solid green and red curves here correspond acording to the standard approach, in order to describe the “diffusion” processes, one can calculate the time dependency of the belt radius (position of the histogram maximum) $r_{\max } (t)$ and the effective width of the belt (standard deviation of the histogram) $\Delta (t) = std(h(r;t))$. An overlap of these two values and complete time-radius depending histogram (colormap of the value $h(r;t)$) is plotted in Fig. 10c.

It is seen directly from this plot that the expansion of this distribution on inhomogeneous substrate reminds an ordinary diffusion. Maximally probable distance from the origin $r_{\max } (t)$ grows with the time slower than linearly: $r_{\max } (t) \sim t$. One can associate some effective exponent with this dependence $r_{\max } (t) \sim t^{\kappa }$, where from the curve convexity, one can expect that $\kappa < 1$. Normally, such an “anomalous diffusion” is called ***sub-diffusion***. In reality, such diffusion never can be described by the unique scaling exponent $\kappa_{0} = const$. Well accepted approximation is still almost scaling behavior with slowly depending on time effective exponent $\kappa = \kappa (t)$. To extract it from the numerical experiment, one can interpolate digitally obtained data for the logarithm $\log (r_{\max } (t))$ and numerically differentiate it by logarithmic variable $\log (t)$.

The dynamic process of the calculation $\kappa (t) = \partial \log (r_{\max } (t))/\partial \log (t)$ is also shown in the Supplementary movie 4, together with the physical expansions of all the described above distributions.

Effective (time depending) diffusion exponent is defined by the relation $r_{\max } = r_{0} t^{{\kappa_{eff} (t)}}$, or in logarithmic scale by the time-depending derivative $\kappa_{eff} (t) = \partial \log (r_{\max } )/\partial \log (t)$, depicted by bold dashed green curve in the subplot (d). The solid green and red curves here correspond to the same dependencies as shown in Fig. 10c, but plotted in log/log scale. This procedure gives finally effective time-depending exponent $\kappa (t) = \partial \log (r_{\max } (t))/\partial \log (t)$, which is plotted in Fig. 11d by dashed green line. It is seen directly from the static plot Fig. 11d and movie that the effective exponent $\kappa (t) = \partial \log (r_{\max } (t))/\partial \log (t)$ completely lies inside intuitively expected interval $0.5 \le \kappa (t) \le 1$. Starting from some transient stage at the very beginning of the process, it attracts to the value $\kappa (t) \approx 0.5$, corresponding to normal diffusion, later one can observe a crossover $\kappa (t) \to 1.0$.

One can observe that asymptotically, at extremely long times, this value tends to the ballistic limit $\kappa (t) \simeq 1$. Physically, it means that almost all the realizations find the orientations of their bodies and velocities corresponding to practically stable running along the “channels”. Such a motion is more or less the same, as it should be in the uniform space. Such asymptotic behavior is probably specific for the motion in model regular potential and would be impossible on more realistic (and complex) fractal reliefs. However, one can expect it at least in any kind of the symmetric potential (with hexagonal or rhombic symmetry, for example), where it is possible to find the ways of moving along fixed direction during long (or even almost infinite) time intervals.

## Conclusions

In this study, we developed numerical model helping us to study the distribution of artificial miniature robots that use actuation in combination with frictional anisotropy in 2D space on an uneven terrain. We show that at extremely long times, these systems tends to behave according to the rules of ballistic diffusion so that their motion tends to be associated with the “channels” of the patterned substrate. Such a motion is more or less the same as it should be in the uniform space. Such asymptotic behavior is specific for the motion in model regular potential and would be impossible on more realistic fractal reliefs. One of the most important conclusions is that to get efficiently propagating “robot” one has to adjust properly frequency of the oscillations and anisotropy of friction. However, such an adjustment can be found only “experimentally” and it is different for different terrains. One has to note that for the practical use, an increasing of the distance from the beginning of motion should be treated as the desirable in some cases, while in the other cases a tendency to follow specific routes (large roads) inside the potential valleys can be treated as the mostly preferable. In this sense, the paper presents a kind of tool which can be used later to find desirable solutions for a number of various practical requirements.

## Supplementary Information

Supplementary Legends.

Supplementary Video 1.

Supplementary Video 2.

Supplementary Video 3.

Supplementary Video 4.

## Data Availability

The datasets used and/or analysed during the current study available from the corresponding author on reasonable request.
