# Supplementary material for: Numerical model of the locomotion of oscillating ‘robots’ with frictional anisotropy on differently-structured surfaces
Source: Sci Rep. 2024 Aug 24;14:19693. doi: 10.1038/s41598-024-70578-1 (PMC11344841; doi:10.1038/s41598-024-70578-1)
Supplement: Supplementary file 1 — Supplementary Legends. [file 41598_2024_70578_MOESM1_ESM.doc]

**Legends for supplementary videos**

Movie 1. Numerically generated potential and basic properties of individual movable automata (see also Figure 2). Heavy “head” with high friction is shown by the large black circle. Small pink circle represents light “tail” with much smaller friction. These two ends are connected by a gray line (“body”) with oscillating length. Due to the difference in mass and friction coefficient $m_{1}>m_{2}$,$\gamma_{1}>\gamma_{2}$, at every oscillation cycle, the common center of mass shifts into the direction from the tail to head. Potential relief (shown by the colormap) causes additional forces, which lead to the change of the motion direction. As a result, a complex curved trajectory appears. In the complex potential relief, trajectories of two originally close realizations of the system mutually deviate.

Movie 2. Simultaneous generation of the Figs. 3 and 4 is reproduced in dynamics

Movie 3. Instant distribution of the automata, their velocities and comet tails trajectories found at time moment $t_{1}>\tau$ starting from a common center with random (uniform in average) distribution of the initial body orientations. The subplots at the right hand side show the positions of the heads and the trajectories, obtained at relatively late time moment $t_{2}>>t_{1}$ for the weak, intermediate and strong potentials, respectively (see also Figure 8).

Movie 4. **Right panel:** Distributions of the body orientation of automata in space and directions of their motion obtained after long time run in the intermediate potential. Distribution of the distances between current head positions and their initial positions is plotted as a probability histogram. See also Figure 9. **Middle panel:** Mutual correlation between evolution of the spatial distribution of the movable automata with random initial distribution of the orientations and statistical properties of the diffusion at different stages of the process. See also Figure 10. **Left panel:** Colormaps of the time evolution of the probability histograms automata (bodies orientation in space and directions of their motion accumulated during sufficiently long time run in the intermediate potential. See also Figure 11.
